# Potential ocular health benefit of short-term omega-3 fatty acids supplementation on the ocular tear film: An observational study

**DOI:** 10.1097/MD.0000000000046566

**Published:** 2026-05-12

**Authors:** Meznah S. Almutairi, Essam S. Almutleb, Abdulrahman A. Bajsair, Gamal A. El-Hiti, Basal H. Altoaimi, Mansour Alghamdi, Saud A. Alanazi, Ali M. Masmali

**Affiliations:** a Department of Optometry, College of Applied Medical Sciences, King Saud University, Riyadh 11433, Saudi Arabia.

**Keywords:** noninvasive tear break-up time, omega-3 supplementation, standard patient evaluation of eye dryness questionnaire, tear ferning, tear film

## Abstract

Omega-3 fatty acids can reduce inflammation and, as a result, decrease the production of inflammatory mediators. The study aimed to evaluate the short-term effects of omega-3 fatty acid supplementation on the tear film. Fifty subjects aged 18 to 27 years were recruited. All subjects received 2 soft gels of molecularly distilled omega-3 fatty acids for 3 consecutive days. A control age-matched group of 50 subjects was included for comparison. The standard patient evaluation of eye dryness (SPEED) questionnaire was completed, followed by the noninvasive tear breakup time (NITBUT), tear meniscus height (TMH), and tear ferning (TF) tests. The first measurements were taken before the supplement was consumed, and the second measurements were taken 24 hours after the third dose of omega-3 fatty acids was administered. The control group subjects did not get omega-3, with measurements on days 1 and 4. Significant (Wilcoxon signed-rank test) differences were found in the median scores of the SPEED (*P* <.001), NITBUT (*P* <.001), and TF (*P* = .040) before and after the consumption of omega-3 fatty acids. However, consuming omega-3 fatty acids showed no significant difference in TMH score. For the control group, no significant differences were observed in the SPEED (*P* = .093), NITBUT (*P* = .149), TMH (*P* = .831), and TF (*P* = .567) scores between days 1 and 4. The oral consumption of omega-3 fatty acids over 3 consecutive days significantly improved the comfort, stability, and quality of the ocular tear film. However, no significant change was observed in tear volume after the consumption of omega-3 fatty acids. Further research is still necessary to verify the general applicability of the findings and to address the existing limitations.

## 1. Introduction

The tear film is the outer part of the ocular surface. It lubricates and protects the ocular surface. It maintains a smooth surface for light refraction. The tear film has a volume of 3 to 10 μL, a thickness of 3 μm, and a pH of approximately 7.45. For simplicity, it consists of 3 major layers. The inner mucin, middle aqueous, and outer lipid layer. Tears are produced by the main lacrimal glands and are drained through the lacrimal puncta.^[1–3]^ Basal tears, reflex tears, and closed-eye tears are known. Basal tears sustain ocular comfort and provide essential nutrients. Reflex tears are produced when the eyes are irritated, while closed-eye tears moisturize the eyes during sleep. Although each type has its unique biochemistry, they all maintain relatively consistent osmolarities.^[4]^

Noninvasive tear break-up time (NITBUT) is a measurement used to assess the stability of the tear film. A decreased TBUT (<10 seconds) is abnormal and often seen in evaporative dry eye disease.^[5]^ Tear osmolarity ranges between 300 and 310 mOsm/Kg in normal eyes and can reach 360 mOsm/kg in dry eye disease.^[6,7]^ Tear hyperosmolarity can lead to the production of inflammatory mediators and damage to the corneal surface and goblet cells. Ocular surface staining and Schirmer test can also be used to evaluate tear production and assess ocular inflammation.^[8,9]^ The assessment of tear meniscus height (TMH) is another method for determining the volume of a tear. The phenol red thread test measures tear secretion.^[10]^ Other tests, such as tear evaporation rate and tear ferning (TF) tests, along with questionnaires, e.g., standard patient evaluation of eye dryness (SPEED) questionnaire, have been used to detect eye dryness.^[11–14]^

The prevalence of dry eye is increasing and becoming a significant public health concern. It affects both the quality of life and visual function and has a significant socio-economic impact. Dry eye involves instability of the tear film, increased osmolarity, and inflammation of the ocular surface. Treatment should restore the homeostasis of the ocular surface system, taking into account factors such as tear film instability and inflammation. The treatment should be long-lasting and personalized, and patients should be educated about the natural history of the disease and the need for potential adjustments in treatment.^[15]^

Omega-3 fatty acids have several carbon-carbon double bonds. They are found in flaxseed, fish, and oil. There are several types of omega-3 fatty acids, but most scientific research focuses on 3 main ones: α-linolenic acid (ALA), eicosapentaenoic acid (EPA), and docosahexaenoic acid (DHA).^[16]^ ALA contains 18 carbon atoms, while EPA and DHA are considered long-chain omega-3 fatty acids because they contain 20 and 22 carbons, respectively (Fig. S1, Supplemental Digital Content, https://links.lww.com/MD/Q904).

Microalgae can synthesize DHA and EPA. When fish consume phytoplankton that feed on microalgae, they accumulate omega-3 fatty acids in their tissues.^[17,18]^ Omega-3 fatty acids serve as components of the phospholipids that make up cell membranes.^[19]^ DHA is found in high quantities in the brain and retina. The treatment of severe dry eyes has been shown to be effective with omega-3 fatty acids.^[20]^ It is believed that dietary adjustments involving omega-3 fatty acids can alleviate symptoms and signs of dry eye syndrome by modulating inflammation on the surface of the eye and enhancing tear-lipid profiles.^[21]^ Diets containing high levels of long-chain omega-3 fatty acids have been recommended for their potential long-term benefits in managing dry eye disease and age-related macular degeneration.^[22,23]^ The total daily amount of EPA and DHA consumed should be no more than 3 G/day, with no more than 2 G/day coming from supplements.

Consuming omega-3 fatty acids can reduce the likelihood of developing cancer, heart disease, and autoimmune disorders.^[24]^ Studies on animals have shown that daily supplementation with omega-3 can effectively treat dry eyes.^[25]^ A combination of topical omega-3 and hyaluronic acid may be more effective than artificial tears.^[26]^ Omega-3 fatty acids in fish lower the occurrence of dry eye.^[27]^ A higher consumption of omega-3 fatty acids compared to omega-6 fatty acids decreases the risk of experiencing dry eyes.^[28]^

Observational studies alone cannot prove causality due to the complex confounding factors that affect the development of dry eye. Therefore, randomized controlled trials are necessary to determine whether omega-3 fatty acid supplementation is effective in treating dry eye. Such trials are important because omega-3 fatty acids may be a low-risk and cost-effective treatment for dry eye syndrome. Various controlled trials of omega-3 fatty acids have been reported, yielding varying results and discordant conclusions. Therefore, the current study aimed to evaluate the short-term impact of omega-3 fatty acids supplementation on ocular tear film comfort, stability, volume, and quality using the SPEED questionnaire, EASYTEAR View+, and TF test.

## 2. Methods

### 2.1. Subjects

The current observational study included 50 subjects (24 males and 26 females), ranging in age from 18 to 27 years (mean standard deviation = 21.9 ± 1.7 years) and with no eye diseases or disorders, who were randomly recruited from students at the College of Applied Medical Sciences in Riyadh. A control group of 50 subjects (25 males and 25 females), aged between 18 and 28 years (21.5 ± 2.3 years), was included for comparison. The sample size was calculated to be 50 participants, with a 95% confidence level and a 5% margin of error. The research was approved by King Saud University’s Institutional Review Board (E-24-8818). Participants signed the informed consent form prior to the commencement of the research. All subjects received 2 soft gels of molecularly distilled omega-3 fatty acids (200 soft gels; Now Foods) for 3 consecutive days. Each capsule contained 180 mg of EPA and 120 mg of DHA.

The SPEED questionnaire was completed first, followed by the NITBUT, TMH, and TF tests. The tests were performed twice on each subject’s right eye. The measurement was carried out for the first time before the supplement was consumed, and a second set of measurements was taken 24 hours after the third dose of omega-3 fatty acids was administered. A 5-minute gap was allowed between tests. The same examiner performed the tests under controlled conditions (e.g., temperature and humidity). The examiner was blinded to whether the subjects were in the study or control groups, as well as to the timing of omega-3 intake and the measurement order, to reduce observer bias.

### 2.2. The SPEED questionnaire

It is used to monitor dry eye symptoms over time, assessing their frequency and severity.^[14,29]^ A score between 5 and 7 and above 8 indicates moderate and severe dry eye symptoms, respectively.

### 2.3. The NITBUT and TMH tests

EASYTEAR View + was used to measure the NITBUT and TMH scores. The NITBUT is the duration, measured in seconds, from the end of a blink to the beginning of the formation of a dry spot in the tear film. If the NITBUT value is <10 seconds, it confirms the presence of dry eye symptoms. The TMH test measures the volume of the tear film and the height of the triangular-shaped cross-section between the margin of the lower lid and the cornea, expressed in millimeters. If the measurement is lower than 0.2 mm, it indicates a dry eye. The NITBUT and TMH scores were measured 3 times, and the mean was recorded.

### 2.4. The TF test

The TF test involves collecting a small tear sample (1 μL) from the lower meniscus of the right eye using a glass capillary tube (10 μL). Each tear sample was dried for 10 minutes at 22 °C and a humidity of <20%. An Olympus DP72 digital microscope will be used to observe and capture the TF images (magnification power of 10×). The TF patterns will be graded based on the 5-point TF grading scale. A TF grade ≥ 2 is considered indicative of dry eye.

### 2.5. Statistical analysis

The SPSS software (version 22, Chicago) was utilized to analyze the data. The data were determined to be non-normally distributed using the Kolmogorov–Smirnov test (*P* <.050). The Wilcoxon signed-rank test was used to analyze the data before and after consumption of omega-3 fatty acid supplementation. Spearman correlation coefficient (*r*) was used to test the association between various parameters. The data were not normally distributed, and the average scores were represented as the median and interquartile range (IQR).

## 3. Results

No significant difference (Wilcoxon signed-rank test) in age was found between male and female subjects in both the study (*P* = .497) and control (*P* = .059) groups. The median (interquartile range; IQR) scores for SPEED, NITBUT, TMH, and TF in the study (N = 50) before and after the consumption of omega-3 fatty acids supplementation are shown in Table 1. Table 2 presents the median (IQR) scores for tear film parameters in the control group on days 1 and 4, when no omega-3 fatty acids were administered.

**Table 1:** Median (IQR) scores for the SPEED, NITBUT, TMH, and TF in the study group (N = 50) before and after 3 consecutive days of omega-3 supplementation.

Parameter	Before omega-3 consumption	After omega-3 consumption	*P*-value
SPEED*	6.5 (0.7)	5.0 (6.0)	<.001
NITBUT (s)*	6.8 (4.0)	8.2 (4.1)	<.001
TMH (mm)	0.21 (0.04)	0.21 (0.03)	.130
TF*	1.4 (0.6)	1.3 (0.8)	.040

* Significant difference (Wilcoxon signed-rank test).

IQR = interquartile range, NITBUT = noninvasive tear breakup time, SPEED = standard patient evaluation of eye dryness, TF = tear ferning, TMH = tear meniscus height.

**Table 2:** Median (IQR) scores for the SPEED, NITBUT, TMH, and TF in the control group (N = 50) on days 1 and 4 with no omega-3 intake.

Parameter	Before omega-3 consumption	After omega-3 consumption	*P*-value
SPEED	6.0 (4.8)	6.0 (4.0)	.093
NITBUT (s)	8.6 (3.5)	8.5 (3.5)	.149
TMH (mm)	0.19 (0.05)	0.18 (0.08)	.831
TF	1.2 (0.9)	1.2 (0.9)	.567

IQR = interquartile range, NITBUT = noninvasive tear breakup time, SPEED = standard patient evaluation of eye dryness, TF = tear ferning, TMH = tear meniscus height.

Significant (Wilcoxon signed-rank test) differences were observed in the median scores of SPEED (*P* <.001), NITBUT (*P* <.001), and TF (*P* = .040) in the study subjects before and after omega-3 supplementation. Conversely, no significant difference (*P* = .130) was found in the median TMH score before and after omega-3 intake. For the control group, no significant differences were observed in the SPEED (*P* = .093), NITBUT (*P* = .149), TMH (*P* = .831), and TF (*P* = .567) scores between days 1 and 4.

No significant differences in age were found between male and female subjects in both the study and control groups (Wilcoxon signed-rank test; *P* = .066). Additionally, there were no significant differences between the control and study groups in TF1 (*P* = .419), TF2 (*P* = .498), NITBUT2 (*P* = .666), SPEED1 (*P* = .822), and SPEED2 (*P* = .159). However, statistically significant differences were observed in NITBUT1 (*P* = .008), TMH1 (*P* = .013), and TMH2 (*P* = .004) between the study and control groups.

Based on the SPEED scores, tear film comfort improved after omega-3 consumption in 31 out of 50 subjects (62%). Meanwhile, the SPEED scores remained unchanged in 9 subjects (18%) and decreased in 20% of the study group after consuming omega-3 fatty acids. For the NITBUT test, tear film stability improved in 40 subjects (80%), while it decreased in 9 individuals (18%) after consuming the supplement. The TF grades of the dried tears indicated an improvement in tear quality in 29 (58%) and a reduction in 16 subjects (32%) after the consumption of omega-3 supplementation. On the other hand, the TF grade remained unchanged in only 5 subjects (10%) after having omega-3 fatty acids.

**Figure 1.**
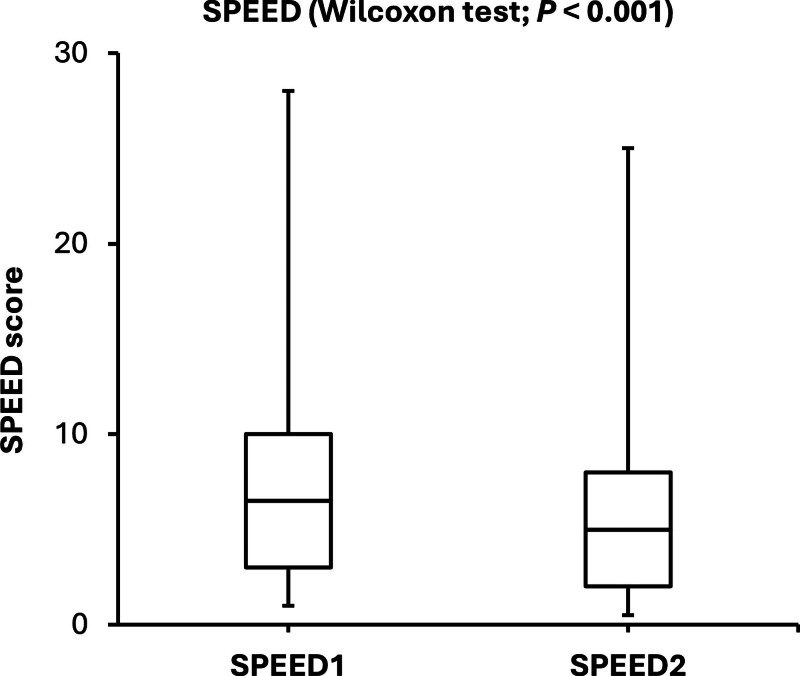
Side-by-side boxplots of the SPEED scores before and after the consumption of omega-3 fatty acids for 3 consecutive days. SPEED = standard patient evaluation of eye dryness.

**Figure 2.**
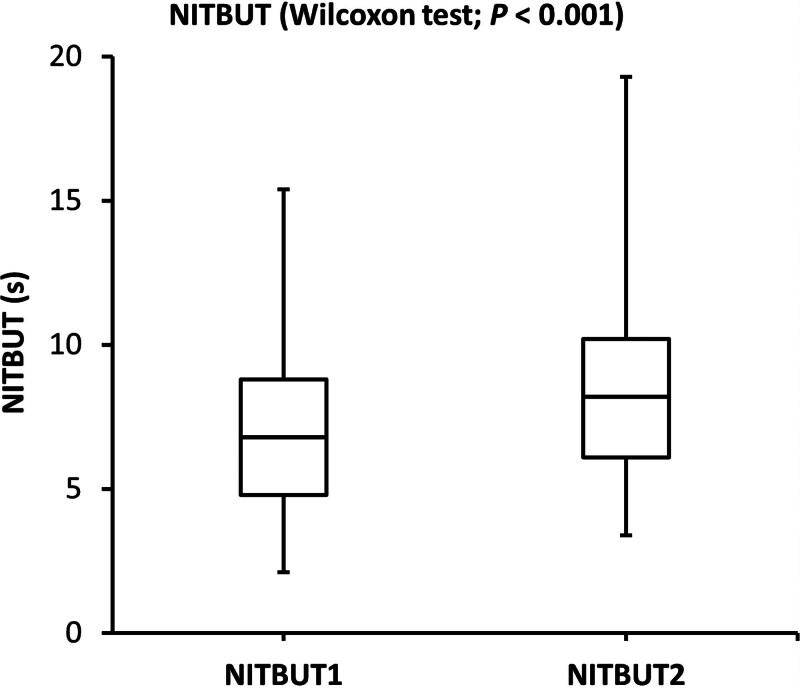
Side-by-side boxplots of the NITBUT scores (s) before and after the consumption of omega-3 fatty acids for 3 consecutive days. NITBUT = noninvasive tear breakup time.

**Figure 3.**
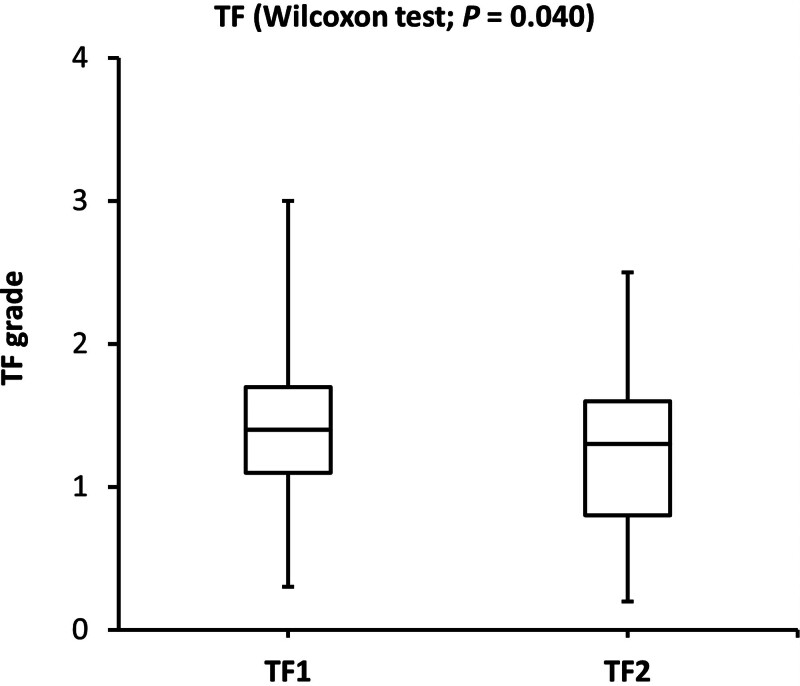
Side-by-side boxplots of the TF grades before and after the consumption of omega-3 fatty acids for 3 consecutive days. TF = tear ferning.

The SPEED and TF scores were significantly lower after the consumption of omega-3 fatty acids. On the other hand, the NITBU scores were significantly higher after consuming omega-3 fatty acid supplementation. The changes in SPEED, NITBUT, and TF scores indicated that comfort, tear film stability, and tear quality were significantly improved after consuming omega-3 fatty acids for 3 consecutive days. The side-by-side boxplots for the SPEED, NITBUT, and TF in the study group (before and after the consumption of omega-3 fatty acids supplementation) are shown in Figures 1–3.

**Figure 4.**
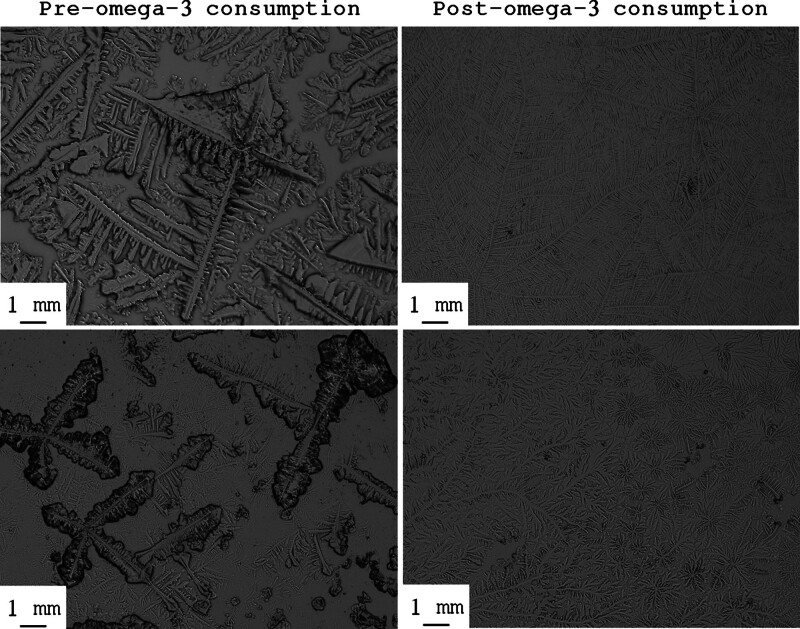
TF images from the same subjects (N = 2) before (A and C) and after (B and D) the consumption of omega-3 fatty acids for 3 consecutive days.

The TF images from the same subjects (N = 2) before and after the consumption of omega-3 fatty acids supplementation are shown in Figure 4. The images of dried tears taken after consuming omega-3 fatty acids display densely packed, finer ferns without gaps between the branches. Such results indicate that the tears have become healthier and of improved quality. In contrast, the images of dried tears before taking omega-3 fatty acids show gaps between the ferns, suggesting lower-quality tears.

The Spearman rho correlation coefficient (*r*) was strong between the SPEED (*R* = 0.784; *P* = .020) and NITBUT scores (*R* = 0.654, *P* <.010) before and after the consumption of omega-3 fatty acids. A medium correlation (*R* = 0.363; *P* = .020) was found between the TMH score before and after the consumption of omega-3 fatty acids. A weak correlation (*R* = 0.289; *P* = .040) was found between the TF grades before and after the consumption of omega-3 fatty acids. For the control group, a strong correlation was found between SPEED (*R* = 0.938, *P* <.010), NITBUT (*R* = 0.990, *P* <.010), and TF (*R* = 0.948, *P* <.010) scores from day 1 to day 4. A weak correlation (*R* = 0.328; *P* = .020) was found in the TMH score between days 1 and 4.

In the study group, no significant differences were found in TF1 (Kruskal-Wallis test; *P* = .070), TF2 (*P* = .611), NITBUT1 (*P* = .052), NITBUT2 (*P* = .581), TMH1 (*P* = .090), TMH2 (*P* = .543), SPEED1 (*P* = .501), and SPEED2 (*P* = .601) between male and female subjects.

## 4. Discussion

The current study examined the relationship between tear film characteristics and the consumption of omega-3 fatty acids, as well as their effect on eye health. The results suggest that omega-3 fatty acids have a beneficial effect on the comfort, stability, and quality of the tear film. Consuming omega-3 fatty acids for a short period (3 consecutive days) is associated with a notable decrease in the SPEED scores and TF grades, as well as an increase in the NITBUT scores. However, no significant change was observed in tear volume after the intake of omega-3 fatty acids.

Omega-3 fatty acids can be considered an effective way to manage dry eye syndrome. They can have a therapeutic effect on the tear film and dry eye syndrome by suppressing ocular tissue inflammation.^[18]^ Eye drops containing omega-3 fatty acids may help alleviate symptoms of dry eye. Omega-3 fatty acids can be combined with anti-inflammatory agents for more effective management of dry eye. For example, omega-3 fatty acids combined with cyclosporine A were found to be more effective in improving TBUT.^[30]^

The current results agree with the meta-analysis from several studies.^[31,32]^ Omega-3 fatty acids consumption led to a significant improvement in the scores obtained from the TBUT (*P* = .007) and Schirmer tests (*P* = .001).^[31]^ However, no significant difference (*P* = .090) was observed in the scores from the ocular surface disease index (OSDI).^[31]^ For example, dry eye subjects (N = 33; 45–90 years) received 2 omega-3 capsules daily (each capsule contains 180 mg EPA and 120 mg DHA) for a month and showed significant improvements (*P* <.05) in the OSDI, Schirmer-I, TBUT, and fluorescein scores.^[33]^ Omega-3 oral nutritional supplements that were administered to 9 subjects 2 weeks before refractive error surgery and 1 month after photorefractive keratectomy led to decreased epithelial healing time and improved TBUT.^[34]^

Another study found that using eye drops containing omega-3 fatty acids at least 3 times a day for 12 weeks improved OSDI and TBUT scores.^[35]^ In addition, the lipid layer thickness was significantly increased (*P* <.001) after 15 minutes of instillation with eye drops containing omega-3 fatty acids and was maintained at 1 hour post-instillation.^[36]^

The benefit of omega-3 fatty acids for long durations has been assessed among dry eye subjects (N = 470).^[37]^ The results from consuming omega-3 fatty acids for 3 and 6 months were similar to those obtained in the current study. The tear film stability (TBUT score) and tear volume (Schirmer score) were significantly increased (*P* <.001) in dry eye subjects who received 4 capsules (each containing 180 mg of EPA and 120 mg of DHA) twice daily for up to 6 months compared to the placebo group. In addition, a significant reduction in dry eye symptoms and tear osmolarity was observed following the consumption of omega-3 fatty acids for this prolonged duration.^[37]^ However, the dose used in this study was 4 times higher than the one used in the current study.

In laboratory settings, omega-3 fatty acids can be converted into powerful anti-inflammatory agents such as protectins and resolvins.^[37]^ These medications help reduce inflammation by inhibiting the production of multiple proinflammatory cytokines in various parts of the body, including the eyes.^[38]^ In an in vivo study, oral supplementation with omega-3 fatty acids has been shown to improve tear stability, thereby enhancing the quality of the oily layer and reducing tear evaporation.^[39]^ Furthermore, omega-3 fatty acids alleviate symptoms of dry eyes by revitalizing the lipid layer in the tear film, addressing meibomian gland dysfunction, and enhancing tear production from the lacrimal gland.^[40]^

Omega-3 fatty acids can help reduce triglyceride levels, although their exact mechanism of action is not fully understood. They are believed to lower triglycerides by suppressing lipogenic gene expression, promoting beta-oxidation of fatty acids, increasing lipoprotein lipase activity, and affecting overall body lipid balance accretion.^[41,42]^ Omega-3 fatty acids are believed to help lower high triglycerides by influencing overall body fat buildup. Multiple studies indicate that consuming omega-3 fatty acids for over 6 weeks can increase the metabolic rate and decrease total body fat.^[43,44]^ Omega-3 fatty acids can reduce inflammation and influence the lipid composition produced by the epithelial cells in the meibomian gland.^[45]^ They also relieve dry eye symptoms, especially when taken in high doses over a long period and with increased EPA levels. However, it is essential to exercise caution when applying these findings broadly, given variations in study outcomes and the diverse characteristics of patients. In summary, it is still recommended to use omega-3 fatty acid supplements for managing dry eye in clinical settings.

It is important to acknowledge the limitations of the current study. The participants were young university students, and the sample size was relatively small. Additionally, a few tests were used to evaluate the tear film parameters, and only 1 type of omega-3 fatty acids supplementation was utilized to assess its impact on the tear film parameters. Moreover, the control group did not receive a placebo intervention. Such awareness could influence their responses on subjective measures (e.g., SPEED score), introducing a possible performance bias. Therefore, a more comprehensive study with a larger and more diverse sample is necessary in the future to gain a better understanding of the impact of omega-3 fatty acids supplementation on the ocular tear film parameters.

## 5. Conclusions

The oral consumption of omega-3 fatty acids over 3 consecutive days significantly improved the comfort, stability, and quality of the ocular tear film. However, no significant change was observed in tear volume after the consumption of omega-3 fatty acids. Further research is still necessary to verify the general applicability of the findings and to address the existing limitations.

## Author contributions

**Conceptualization**: Essam S. Almutleb, Gamal A. El-Hiti.

**Data curation**: Meznah S. Almutairi, Essam S. Almutleb, Gamal A. El-Hiti, Basal H. Altoaimi, Mansour Alghamdi, Saud A. Alanazi, Ali M. Masmali.

**Formal analysis**: Meznah S. Almutairi, Essam S. Almutleb, Gamal A. El-Hiti, Basal H. Altoaimi, Mansour Alghamdi, Ali M. Masmali.

**Funding acquisition**: Meznah S. Almutairi, Gamal A. El-Hiti.

**Investigation**: Abdulrahman A. Bajsair, Gamal A. El-Hiti.

**Methodology**: Meznah S. Almutairi, Essam S. Almutleb, Abdulrahman A. Bajsair, Gamal A. El-Hiti.

**Project administration**: Gamal A. El-Hiti.

**Resources**: Gamal A. El-Hiti.

**Software**: Meznah S. Almutairi, Essam S. Almutleb, Gamal A. El-Hiti, Basal H. Altoaimi.

**Supervision**: Gamal A. El-Hiti.

**Validation**: Meznah S. Almutairi, Essam S. Almutleb, Gamal A. El-Hiti, Basal H. Altoaimi, Mansour Alghamdi.

**Visualization**: Meznah S. Almutairi, Essam S. Almutleb, Gamal A. El-Hiti, Basal H. Altoaimi, Mansour Alghamdi.

**Writing – original draft**: Gamal A. El-Hiti.

**Writing – review & editing**: Gamal A. El-Hiti, Saud A. Alanazi, Ali M. Masmali.

## Supplementary Material
