# Supplementary material for: Potential ocular health benefit of short-term omega-3 fatty acids supplementation on the ocular tear film: An observational study
Source: Medicine (Baltimore). 2026 May 12;104(49):e46566. doi: 10.1097/MD.0000000000046566 (PMC12688933; doi:10.1097/MD.0000000000046566)
Supplement: Supplementary file 1 [file medi-104-e46566-s001.docx]

**Supplementary Materials**

**Potential ocular health benefit of short-term omega-3 fatty acids supplementation on the ocular tear film: An observational study**

Meznah S. Almutairi, PhD,† Essam S. Almutleb, PhD,† Abdulrahman A. Bajsair, OD, Gamal A. El-Hiti, PhD*, Basal H. Altoaimi, PhD, Mansour Alghamdi, PhD, Saud A. Alanazi, PhD, Ali M. Masmali, PhD

*Department of Optometry, College of Applied Medical Sciences, King Saud University, P.O. Box 10219, 11433 Riyadh, Saudi Arabia*

† Contributed equally to this work

** Correspondence: Gamal A. El-Hiti, Department of Optometry, College of Applied Medical Sciences, King Saud University, P.O. Box 10219, 11433 Riyadh, Saudi Arabia (e-mail: gelhiti@ksu.edu.sa)*





**Figure S1.** Chemical structures of ALA, EPA, and DHA.
